# Acute Angle‐Closure Glaucoma Secondary to Vitreous Prolapse Following Ocular Trauma

**DOI:** 10.1155/crop/9483412

**Published:** 2026-05-24

**Authors:** Cullen Moran, So Yeon Uhm, Fitz Gerald I. Diala, Vaama Patel, Sylvia Groth

**Affiliations:** ^1^ Vanderbilt Eye Institute, Nashville, Tennessee, USA, vanderbilthealth.com

**Keywords:** angle closure, glaucoma, trauma, vitreous

## Abstract

Secondary acute angle‐closure glaucoma (AACG) caused by vitreous prolapse in a phakic patient is exceedingly rare. We report a case of AACG secondary to vitreous prolapse, occurring 2 weeks after orbital trauma in a 34‐year‐old male healthcare worker. The patient presented with acute left eye pain, nausea, and vision loss after performing cardiopulmonary resuscitation (CPR) at his workplace. Two weeks earlier, he sustained a nonoperative orbital floor fracture and commotio retinae from a motor vehicle accident. Examination revealed decreased vision and an intraocular pressure (IOP) of 59 mmHg. Vitreous prolapse into the anterior chamber was detected. Urgent anterior vitrectomy was performed, leading to normalization of IOP and visual acuity improvement to 20/50 at 2 months postoperatively. Valsalva maneuvers and repetitive head movements during CPR likely precipitated vitreous herniation through trauma‐induced zonular loss. This case underscores the need to consider vitreous prolapse in the differential for acute angle closure, especially in patients with recent ocular trauma.

## 1. Introduction

Vitreous prolapse leading to acute IOP elevation has been reported sparsely in the literature. One such case was reported in a pseudophakic patient with ocular trauma resulting in posterior capsular rupture and subsequent vitreous prolapse with secondary angle closure [1]. Other cases of angle‐closure glaucoma with vitreous prolapse in pseudophakic patients after Nd:YAG capsulotomy have been reported without antecedent trauma [2–6]. There are no reported cases in the literature of vitreous prolapse following orbital trauma in a phakic patient. We present a case of a patient with recent ocular trauma who developed acute glaucoma due to vitreous prolapse after performing chest compressions.

## 2. Case Presentation

A 34‐year‐old male presented to the emergency department after acute onset left eye pain, redness, and vision loss. Two weeks prior to this presentation, the patient had been in a motor vehicle accident and sustained left eye trauma including nonoperative, nondisplaced left orbital floor and moderately displaced orbital roof fractures, as well as commotio retinae of the left eye that were evaluated at an outside hospital. Two weeks after the motor vehicle accident, he was required to perform CPR with chest compressions for several minutes on another person while at work. While performing CPR, he experienced sudden onset of severe left eye pain, redness, and vision loss. He also reported left brow ache and nausea. This prompted his presentation to the emergency department for further evaluation.

On examination, the right eye had normal visual acuity, intraocular pressure, and pupil. His visual acuity in the left eye was counting fingers. His left pupil was irregular and fixed at 6 mm with no response to light. There was a left relative afferent pupillary defect by reverse. His intraocular pressure was 59 mmHg by Goldmann applanation. Extraocular movements were full in both eyes. Confrontation visual fields were full in the right eye, and in the left eye there was global depression.

Slit lamp examination of the right eye was normal. The right eye angles were open to scleral spur in all quadrants on gonioscopy. In the left eye, no angle structures were visible. The left eye had 3+ conjunctival injection and 2+ corneal edema with a hazy view of the anterior chamber, which appeared shallow. There was no evident anterior chamber cell visible through the hazy cornea. Material resembling vitreous was also present in the anterior chamber but the details were somewhat obscured by the corneal edema. The pupil was irregular and middilated at 6 mm. There was trace nuclear sclerosis. There was no obvious lens subluxation. The vitreous was clear. The fundus examination was limited by corneal edema and patient discomfort but did demonstrate two small dot blot hemorrhages superior to the disc and commotio retinae.

Medical treatment was initiated with 500 mg of IV acetazolamide, along with three rounds of topical dorzolamide/timolol every 15 min and three rounds of topical brimonidine every 15 min. Approximately 2 h after initiating medical therapy, the intraocular pressure remained elevated at 50 mmHg. An anterior chamber paracentesis was attempted using a 27‐gauge needle, but no aqueous could be removed. The patient was in persistent, severe, unrelenting pain. He was urgently sent to glaucoma clinic for further evaluation by a glaucoma specialist. An anterior chamber tap was again attempted with a 27‐gauge needle in the clinic, but again, no aqueous could be removed, raising concern for mechanical obstruction. Examination by the glaucoma specialist confirmed the presence of vitreous in the anterior chamber.

The patient was taken urgently to the operating room for anterior vitrectomy. During the case, dilute triamcinolone acetonide was injected into the anterior chamber to visualize vitreous. A large, rounded cloud of vitreous was seen coming from a hole of nasal zonular loss. Nearly the entire anterior chamber was filled with prolapsed vitreous. Anterior vitrectomy was performed. Immediate deepening of the anterior chamber and softening of the eye was noted. The lens was noted to be stable with only focal zonular loss.

The patient′s intraocular pressure was 14 mmHg the day after surgery. He recovered to a pinhole visual acuity of 20/50 by postoperative Month 2. The anterior chamber remained clear of vitreous, and the eye was comfortable.

## 3. Discussion

This case is the first to describe post‐traumatic vitreous prolapse leading to secondary acute angle‐closure glaucoma in a phakic patient. Blunt ocular trauma can induce zonulysis through transient equatorial expansion of the globe with rapid anterior–posterior compression [7]. Experimental studies in eye trauma have demonstrated zonulysis in blunt trauma with impact energy as low as 2 J [8]. Computational models and observational studies have demonstrated a higher propensity for lens deformation in younger phakic patients, which transmits a significant amount of force to the zonules, especially where they insert near the ciliary body [9, 10].

This patient sustained ocular trauma 2 weeks prior to his presentation, with enough force generated to cause an orbital floor fracture and commotio retinae. This trauma also likely led to a focal area of zonular dehiscence. Then, on the day of his angle‐closure event, he had to perform CPR while at work. The repetitive forceful anterior head motion, combined with Valsalva, likely resulted in increased posterior pressure that forced the vitreous through the region of zonular dehiscence, filling the anterior chamber with vitreous.

Several alternative diagnoses were considered in this patient. Lens subluxation or dislocation can produce pupillary block or iridocorneal apposition and may be suggested by phacodonesis, iridodonesis, asymmetric anterior chamber depth, or visible lens edge. Suprachoroidal hemorrhage or effusion can present with a shallow anterior chamber and high IOP, often with severe pain and a characteristic posterior segment configuration on B‐scan ultrasound. Aqueous misdirection classically features a uniformly shallow anterior chamber with a relatively quiet eye and may follow surgery more often than trauma. Traumatic hyphema can elevate IOP through trabecular obstruction and may be apparent clinically or on gonioscopy once corneal clarity permits. When corneal edema or poor visualization limits gonioscopy, ultrasound biomicroscopy (UBM) or anterior segment OCT can help demonstrate vitreous in the anterior chamber/angle, lens position, and anterior chamber configuration, and can aid differentiation from lens subluxation or aqueous misdirection. In our case, visible anterior vitreous and rapid normalization of chamber depth and IOP after anterior vitrectomy supported vitreous‐related secondary angle closure without need for further diagnostic workup.

Vitreous prolapse into the anterior chamber can cause acute IOP elevation by multiple mechanisms. In the case of this patient, a large cloud of vitreous mechanically obstructed the trabecular meshwork. Prolapsed vitreous in combination with anterior displacement of the lens–iris diaphragm can also obstruct passage of aqueous at the pupillary margin, promoting anterior bowing of the iris in a pupillary block‐type mechanism. In this patient, the near‐complete filling of the anterior chamber with vitreous, failure of paracentesis, and immediate chamber deepening and IOP reduction after anterior vitrectomy together support a predominantly mechanical vitreous obstruction mechanism, with possible contribution from vitreous pupillary block.

This patient′s elevated intraocular pressure and severe pain were refractory to topical dorzolamide, timolol, and brimonidine as well as IV acetazolamide. Additional medical management in this situation may include consideration of miotics or cycloplegics. In this case, miotics risk shifting the lens–iris diaphragm anteriorly, possibly worsens the angle‐closure mechanism. Cycloplegics, conversely, may deepen the anterior chamber and reduce the pupillary‐block component by posteriorly rotating the lens–iris diaphragm, although their impact may be limited when the chamber is already densely occupied by vitreous.

Anterior chamber paracentesis as an intermediate interventional measure failed in this patient because of the volume of vitreous that obstructed the paracentesis needle tip. It is worth noting that there are risks inherent to anterior chamber paracentesis in a case like this, including retinal traction and tear, that must be weighed with the goal of temporary relief for the acute pressure elevation. An additional consideration in many cases of angle closure is laser peripheral iridotomy (LPI), which would be expected to benefit primary pupillary block mechanisms. However, in vitreous‐associated secondary closure dominated by mechanical obstruction, LPI is unlikely to provide meaningful relief. Even if a pupillary‐block component is suspected, definitive decompression generally requires clearing the anterior vitreous.

Anterior vitrectomy offered the most direct method to remove the obstruction, reopen the angle, and restore aqueous outflow. Injection of triamcinolone into the anterior chamber at the beginning of the case was a crucial step because it coats vitreous strands, improving visualization of the extent of prolapse and helping confirm complete clearance at the end of vitrectomy. Lens removal was not required because the crystalline lens remained stable with only focal zonular loss, and there was no intraoperative evidence of lens subluxation. In traumatic vitreous prolapse, lensectomy may be indicated when zonular dialysis is extensive, the lens is unstable or contributing to pupillary block, cataract limits visualization, or adequate vitrectomy cannot be performed without compromising lens support. In a young patient with a stable lens, preservation avoids the additional morbidity of lensectomy and secondary IOL planning.

The patient ultimately achieved an acceptable visual outcome, likely limited in part by mild optic nerve compromise and traumatic cataract formation. This case emphasizes the importance of considering vitreous prolapse in the differential diagnosis of acute angle‐closure glaucoma, particularly in patients with a recent history of ocular trauma.

## Funding

No funding was received for this manuscript.

## Conflicts of Interest

The authors declare no conflicts of interest.

## Data Availability

Data sharing is not applicable to this article as no datasets were generated or analyzed during the current study.
